# Functional Variant in the Autophagy-Related 5 Gene Promotor is Associated with Childhood Asthma

**DOI:** 10.1371/journal.pone.0033454

**Published:** 2012-04-20

**Authors:** Lisa J. Martin, Jayanta Gupta, Soma S. S. K. Jyothula, Melinda Butsch Kovacic, Jocelyn M. Biagini Myers, Tia L. Patterson, Mark B. Ericksen, Hua He, Aaron M. Gibson, Tesfaye M. Baye, Sushil Amirisetty, Anna M. Tsoras, Youbao Sha, N. Tony Eissa, Gurjit K. Khurana Hershey

**Affiliations:** 1 Department of Pediatrics, Cincinnati Children’s Hospital Medical Center, University of Cincinnati, Cincinnati, Ohio, United States of America; 2 Department of Medicine, Baylor College of Medicine, Houston, Texas, United States of America; University of Tübingen, Germany

## Abstract

**Rationale and Objective:**

Autophagy is a cellular process directed at eliminating or recycling cellular proteins. Recently, the autophagy pathway has been implicated in immune dysfunction, the pathogenesis of inflammatory disorders, and response to viral infection. Associations between two genes in the autophagy pathway, *ATG5* and *ATG7*, with childhood asthma were investigated.

**Methods:**

Using genetic and experimental approaches, we examined the association of 13 HapMap-derived tagging SNPs in *ATG5* and *ATG7* with childhood asthma in 312 asthmatic and 246 non-allergic control children. We confirmed our findings by using independent cohorts and imputation analysis. Finally, we evaluated the functional relevance of a disease associated SNP.

**Measurements and Main Results:**

We demonstrated that *ATG5* single nucleotide polymorphisms *rs12201458* and *rs510432* were associated with asthma (p = 0.00085 and 0.0025, respectively). In three independent cohorts, additional variants in *ATG5* in the same LD block were associated with asthma (p<0.05). We found that rs510432 was functionally relevant and conferred significantly increased promotor activity. Furthermore, Atg5 expression was increased in nasal epithelium of acute asthmatics compared to stable asthmatics and non-asthmatic controls.

**Conclusion:**

Genetic variants in *ATG5*, including a functional promotor variant, are associated with childhood asthma**.** These results provide novel evidence for a role for *ATG5* in childhood asthma.

## Introduction

Asthma is a chronic, inflammatory disease of the respiratory airways leading to episodes of wheezing, shortness of breath, chest tightness and cough. About 300 million people are affected by asthma globally, with 20 million people in the United States suffering from the condition [1], [2] including 10 million children (13.8%) [3]. Parental asthma is a strong predictor of childhood asthma, suggesting a strong genetic basis [4]. However, genes that have been associated with asthma account for only a minor portion of disease heritability [5] suggesting that undiscovered genetic variants likely exist in understudied pathways relevant to asthma.

Autophagy is a cellular process directed at recycling of cellular proteins and removal of intracellular microorganisms. Though traditionally thought to be a mechanism directed at survival during starvation, evidence suggests that autophagy has a role in innate and adaptive immune responses [6]. In fact, autophagy has been linked to B lymphocyte development [7], antigen presentation [8], and antiviral immunity [9]. More recently, autophagy has been implicated in the lung, with increased autophagy and activation of autophagy proteins in lung tissue from chronic obstructive pulmonary disease patients [10]. In fact, the autophagy pathway has been reported to respond to cigarette smoke exposure and has been postulated to be a key component of the lung tissue injury response to chronic smoke exposure [10], [11]. If bronchial epithelial cells deficient in an autophagy protein are hyperresponsive to methacholine exposure, it is conceivable that autophagy gene dysregulation results in changes in the epithelial factors released; these epithelial factors may then contribute to smooth muscle hyperreactivity in asthmatics.

Given the evidence implicating autophagy in immune responses and inflammation, we examined whether variants in autophagy genes were associated with asthma. We focused on autophagy-related 5 gene (*ATG5)* and *autophagy-related 7 gene (ATG7)* because *ATG5* is essential for autophagosome formation [9], and *ATG7* has been previously shown to be associated with airway hyperresponsiveness in animal models [12]. We hypothesized that *ATG5* and *ATG7* polymorphisms and/or dysregulated expression of these genes are associated with childhood asthma. To test our hypothesis, we genotyped tagging single nucleotide polymorphisms (SNPs) in 312 asthmatic and 246 non-asthmatic non-allergic children and supported our findings using additional cohorts of children and adults. We identified 2 SNPs in *ATG5* associated with asthma, including one in the putative promotor, which we demonstrate to be functionally relevant.

## Methods

### Ethics

The study protocol was approved by the Cincinnati Children’s Hospital Medical Center Institutional Review Board. Parents gave written informed consent for the children’s participation, and children gave their assent.

### Study Populations

The primary analysis cohort included children aged 4–17 years from the greater Cincinnati, Ohio metro area who were enrolled in either the Greater Cincinnati Pediatric Clinic Repository (GCPCR) or the Genomic Control Cohort (GCC) [13], [14]. Due to sample size considerations, analyses were restricted to individuals where self-reported race was white/Caucasian. Asthma cases (N = 312) were derived from the GCPCR, a clinic-based pediatric repository. Asthma was diagnosed according to American Thoracic Society (ATS) guidelines [15]. PFT data was available for 220 children with asthma. Non-asthmatic non-allergic control subjects were derived from both the GCPCR and the GCC, the latter being a population-based cohort representative of the Greater Cincinnati area. Controls had no personal history of allergies or asthma and no family history of asthma (N = 246). For simplicity, this case control cohort is referred to as the GCPCR cohort.

Genetic data from two additional cohorts, the Childhood Asthma Management Program (CAMP) and the Childhood Asthma Research and Education (CARE) studies, were extracted from the database of Genotypes and Phenotypes (dbGaP) (http://www.ncbi.nlm.nih.gov/gap) with permission. Our analysis included 334 family trios of European ancestry from CAMP and 95 trios of European ancestry from CARE with Affymetrix 6.0 genotyping data. In addition to CAMP and CARE, we also evaluated genetic associations in 71 GCC participants with parent-reported asthma and 211 adults from the Cincinnati Control Cohort [16] with no personal or family history of asthma, all of which had Affymetrix 6.0 genotyping data available. These case/control cohorts are referred to together as the “CINCY” cohort for these analyses.

### Selection of SNPs and Genotyping Procedures

For the GCPCR cohort, European descent population (CEU) tagging SNPs were selected based on HapMap NCBI Build 35 (http://www.hapmap.org) using the pair-wise Tagger algorithm (r^2^<0.8, minor allele frequency (MAF)>0.05) [17]. Eight tagging SNPs were identified in *ATG5*, but due to power concerns and low MAF (<0.1), only four tagging SNPs (rs3804329, rs671116, rs12201458, rs573775) were included in the analysis from our custom Illumina GoldenGate assay. Likewise, 10 tagging SNPs were identified in *ATG7*, but 7 (rs1499082, rs2606742, rs2606750, rs346078, rs4684787, rs3856794, rs2305295) were analyzed due to low MAF. Additionally, *ATG5* SNPs located in the 5′ untranslated region (UTR) (rs510432) and 3′ UTR/flanking region (rs1322178) were genotyped. Thirty ancestry informative markers (AIMs) were also genotyped [13]. Genotyping was performed according to manufacturer’s protocol (http://www.illumina.com) and assigned using BeadStudio (V3.2, Illumina, San Diego, CA). Call rates were >99%. All SNPs were in Hardy-Weinberg equilibrium (HWE). Five asthmatic (2 M, 3 F) children were excluded due to missing call rates greater than 20%.

### Gene Expression Studies

Microarray data were derived from previously published data [18]. Briefly, nasal mucosal cells were collected from asthmatic and control participants using a CytoSoft Brush (Medical Packaging Corp, Camarilo, CA). The methods for sample collection, sample processing, RNA isolation, and microarray hybridization using the HG-U133A GeneChip (Affymetrix, Santa Clara, CA) have been described previously [18]. The cells were largely comprised (>92%) of respiratory epithelial cells. Several genes identified using this approach have been implicated in asthma pathogenesis in other studies, validating this approach [19], [20], [21].

**Table 1:** Characteristics of the GCPCR population.

	Asthmatic	Non-allergic
Total children, N	317	246
Children after exclusions, N^a^	312	246
Mean age (years) *±* SD	10.04 *±* 3.44^b^	11.79 *±* 3.40
Male (%)	54.5	49.2
FEV1 (% predicted ± SD)	100.5 ± 14.5	–

a Indicates sample passing quality control.

b Indicates significant differences (p<0.05) with non-allergic control children.

**Table 2:** Genotyped SNPs and their minor allele frequencies (MAF) in the GCPCR cohort.

Gene	SNP	Alleles	Minor Allele	location	Type	MAF -Hapmap CEU	MAF - cases	MAF - controls
*ATG5*	rs1322178	C/T	T	106631781	3′ UTR	0.21	0.22	0.17
	rs12201458	A/C	A	106642688	intron	0.09	0.09	0.16^a^
	rs3804329	A/G	G	106686428	intron	0.22	0.22	0.17
	rs671116	C/T	C	106760598	intron	0.40	0.39	0.33
	rs573775	C/T	T	106764867	intron	0.30	0.32	0.27
	rs510432	A/G	G	106774031	5′ UTR	0.46	0.51	0.42^b^
*ATG7*	rs346078	C/G	C	11302840	intron	0.36	0.35	0.36
	rs2606750	A/G	A	11347151	intron	0.41	0.36	0.36
	rs2606742	C/T	C	11366717	Intron	0.15	0.20	0.17
	rs1499082	A/G	G	11402955	Intron	0.17	0.13	0.15
	rs3856794	C/G	G	11537105	Intron	0.20	0.14	0.15
	rs4684787	C/T	T	11554865	Intron	0.34	0.33	0.32
	rs2305295	A/G	G	11571302	synonymous coding	0.32	0.33	0.34

a p = 0.00085; OR = 0.52, 95% CI, 0.36–0.77.

b p = 0.0025; OR = 1.47, 95% CI, 1.14–1.88.

### Cloning of Atg5 Promotor Fragments and Determination of Promotor Activity

In order to examine the potential impact of rs510432 (located 335bp upstream of the putative human *ATG5* transcription start site) on promotor function, we determined the effect of this SNP on promotor activity. Human genomic deoxyribonucleic acid (DNA) was isolated from peripheral blood mononuclear cells isolated from Buffy coats obtained from local blood bank using Ficoll Hyperpaque method (Stem Cell Technologies). Genomic DNA was subjected to PCR amplification using primers [Fragment 1 Sense AGAGACCTGCTTTCGGCCTG (Location –6081 to –377), Fragment 2 Sense TGCTAATGGCAGTGCATCTCA (Location –4532 to –377), Fragment 3 Sense CAAGTGATGGTTAGGGTTCATGG (Location –3555 to –377) & Fragment 4 Sense TGGAGGAAATGGTAAGGCCAA (Location –2749 to –377), Anti Sense primer GCCCTCCGTGTTCTGCCTAA] designed to generate four fragments containing the Atg5 promotor. Sequence analysis confirmed that the promotor fragments contained the non-variant allele for rs510432. The resulting promotor fragments were purified and sub-cloned into PGL4.20 Firefly Luciferase vector and co-transfected into HEK293 cells along with PGL4.73 Renilla transfection control vector. Briefly, we transfected 0.8 million HEK cells with 2 µg of construct plasmid and 0.08 µg PGL4.73 plasmid (transfection control) simultaneously. This serves as an internal control for transfection efficiency and also eliminates the need for viability testing. After 16 hours, the cells were lysed and Firefly and Renilla Luciferase activities were determined. Promotor activity was assessed using dual Luciferase assay kit according to the manufacturer’s instructions (Promega corp). Site-directed mutagenesis was done using PCR Strategy (Primer Sense: CAACAAAGTAGAGAAGAAGATCAAATGAGAAAATGGATGGGAAAGTACTTTG & Primer Anti-Sense: CAAAGTACTTTCCCATCCATTTTCTCATTTGATCTTCTTCTCTACTTTGTTG) to generate the rs510432 allelic variants and were confirmed by sequencing. In each independent experiment performed, the average value of Firefly luciferase activity and Renilla luciferase activity was calculated. The fold difference was measured using the Renilla luciferase as denominator. PGL4.20 empty vector fold ratio was empirically valued at 1 and rest of the fold ratios were normalized to PGL4.20. The values for each of the three independent experiments were tabulated. The final results are presented as mean with error bars (+/– 1 SD). Paired two tailed student’s t-test was performed to test differences by allelic variant, with an alpha of 0.05 set as statistical significance.

### Analysis

Data were analyzed using SAS (V9.1, SAS Inc., Cary, NC) and PLINK (V1.05; http://pngu.mgh.harvard.edu/purcell/plink/). Differences in age and sex in disease and control groups were tested using t-tests and chi-square tests. To ensure genotyping quality, SNPs exhibiting deviations in HWE (p < 0.0001) were excluded. Population stratification was assessed using EIGENSTRAT and 30 AIMs [22]. There was no evidence of population stratification (λ  =  1), thus no adjustment was applied.

To test for association with asthma, we used logistic regression based on an underlying additive genetic model including age and sex as covariates. To test association with PFTs, we used linear regression based on an underlying additive model and included age and sex as covariates. Results in GCPCR were evaluated after correcting for multiple testing using a Bonferroni adjustment taking into account the average linkage disequilibrium (LD) correlation of 0.239 among 13 SNPs (p-value <0.007) using the freely available Simple Interactive Statistical Analyses Software (http://www.quantitativeskills.com/sisa/). LD was examined using Haploview (V4.1 (http://www.broadinstitute.org/scientific-community/science/programs/medical-and-population-genetics/haploview/haploview). Given the small sample sizes of our additional cohorts (CAMP, CARE, CINCY), we evaluated evidence of association at the nominal level as we were seeking replicate findings from the GCPCR.

### SNP Imputation

Based on HapMap CEU results (release 22), imputation was performed after filtering out SNPs with genotyping call rates < 90%, MAF < 10%, and HWE p-value < 0.0001 with MACH [23]. To ensure data quality, imputed genotypes with r^2^<0.3 were removed [24]. Imputed SNPs were then tested for association with asthma using PLINK. Meta-analysis of the results from imputed SNPs was performed with a weighted Z score (Stouffer’s Z_trend_) as implemented in METAL with the sample weights taken as the square root of the sample size [25]. Evidence of association was evaluated using the same significance threshold as our discovery (GCPCR) cohort.

### Analysis of mRNA Expression

To analyze Affymetrix gene expression, we applied Robust Multi-array Analysis (RMA), in GeneSpring (V7.3.1, Agilent Technologies, Lexington, MA). Briefly, RMA adjusts for chip background, normalizes across chips, and summarizes the data. Expression levels were estimated by using the MicroArraySuite 5.0 algorithm (Affymetrix, Santa Clara, California). Intensities were normalized to the median expression level of the control samples [18]. We then performed a one-way analysis of variance (ANOVA) followed by a Tukey-Kramer post-hoc test (for pairwise statistical significance between the acute asthmatics, stable asthmatics, and non-asthmatics) using PRISM (GraphPad Software Inc., La Jolla, CA).

## Results

### Subject Characteristics

The mean age of asthmatic and non-allergic groups was 10.0 and 11.8 years, respectively (Table 1) with the non-allergic group being slightly, but significantly older. There were no significant differences in sex between cases and controls.

### Genetic Associations between ATG5 and Childhood Asthma

**Figure 1:**
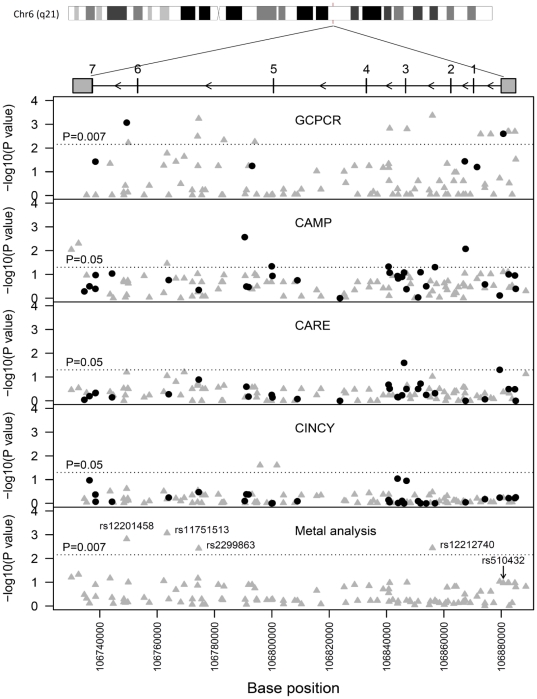
Identification of association between asthma and *ATG5* SNPs. Negative log_10_ p value of the associations in GCPCR, CAMP, CARE, and CINCY cohorts are presented as well as the meta analysis (METAL). Black circles represent genotyped SNPs and grey triangles represent imputed SNPs. The dotted line represents significance (p = 0.007 for GCPCR and METAL after correction for multiple comparisons, p = 0.05 for CAMP, CARE, CINCY).

**Figure 2:**
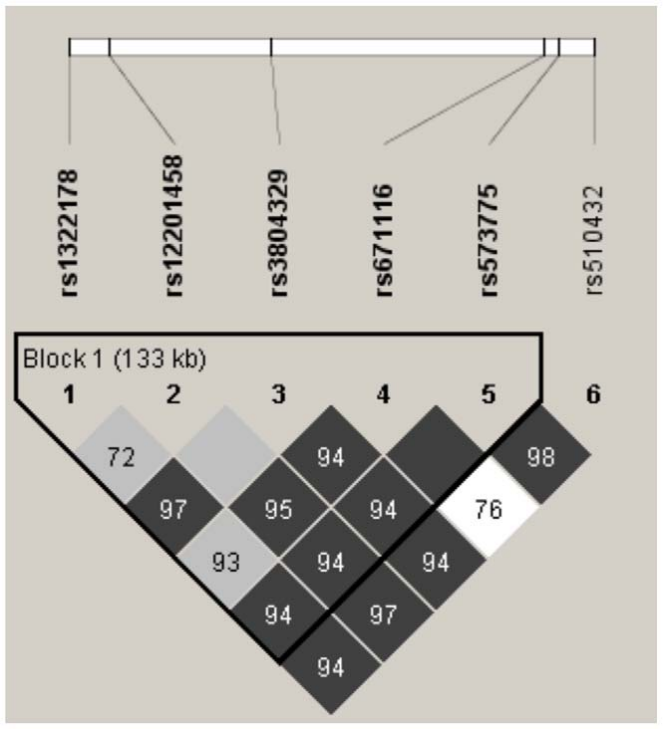
LD plot and identification of haplotype block in the *ATG5* gene. The position of the 6 SNPs within the *ATG5* gene are shown above the plot. *D’* values, indicating extent of LD between SNPs, are noted on the squares. Higher color intensity of the squares indicates higher LD between SNPs. The inverted black triangle represents a single haplotype block (estimated by Gabriel’s 90% bounds on *D’* ^30^).

Two variants in *ATG5* were significantly associated with asthma in the GCPCR (Table 2, Figure 1). The minor allele (A) of *ATG5* rs12201458 was associated with a decreased risk of asthma (p = 0.00085; OR = 0.52, 95% CI, 0.36–0.77), while the minor allele (G) of ATG5 rs510432 was associated with increased asthma risk (p = 0.0025; OR = 1.47, 95% CI, 1.14–1.88). The pattern of LD and haploype block formation within the *ATG5* gene is displayed in Figure 2. Haploview defined one 133 kb haplotype block within the gene region consisting of 5 SNPs using the Confidence Interval method^31^. Interestingly, rs510432 and rs12201458 were not in the same haplotype block. There were no significant disease associations with any of the *ATG7* SNPs examined.

Among asthma cases, we next examined whether *ATG*5 or *ATG*7 SNPs were associated with pulmonary function tests, including FEV1 (% predicted) and FEV1/FVC (% predicted). There was no evidence of association (p>0.38) between *ATG*5 and pulmonary function within the asthmatic group (data not shown). For *ATG*7 SNPs, a single SNP exhibited nominal association (rs2606742, p = 0.04).

To detect additional *ATG5* SNP associations with asthma in the GCPCR cohort, we imputed 112 SNPs located on chromosome 6 between 106734603 and 106885077 base pairs where *ATG5* is located. One hundred and four SNPs passed our imputation criteria and 15 were associated with asthma after correction for multiple comparisons (p<0.007) (Figure 1). Importantly, the MAF in the controls were consistent with HapMap CEU MAF. Further, there was 99% agreement between genotyped SNPs and the corresponding imputed SNPs indicating good imputation quality.

We next examined associations of *ATG5* SNPs with asthma in three additional independent cohorts: CAMP, CARE and CINCY (Figure 1) using available genome-wide SNP data. In each cohort, *ATG5* SNPs were nominally associated with asthma (p<0.05). Further, using meta-analysis, we identified four SNPs (rs12212740, p = 0.0036; rs12201458, p = 0.0015; rs2299863, p = 0.0038; and rs11751513, p = 0.00086) associated with asthma at our multiple testing correction p-value of 0.007. Interestingly, in the meta-analysis, our most strongly associated SNP from the discovery cohort (rs510432) did not exhibit significant evidence of association across the studies. However, failure of this association was driven by the CAMP cohort. If the direction of association was not incorporated into the meta analysis, this SNP would have reached significance after multiple testing correction (Stouffer’s z p = 0.0006).

### Asthma-associated rs510432 SNP G Variant Allele Confers Enhanced ATG5 Promotor Activity

**Figure 3:**
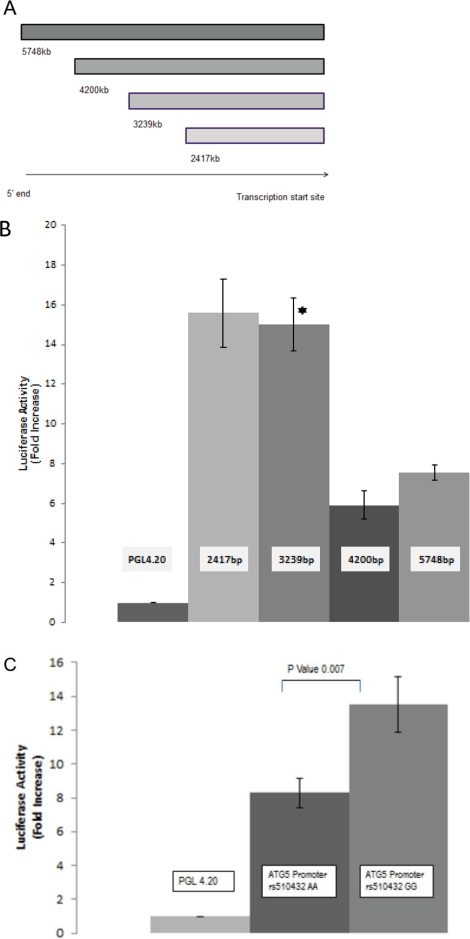
Asthma-associated rs510432 SNP G variant allele confers enhanced promotor activity. A. ATG5 promotor fragments generated from genomic DNA isolated from human peripheral blood mononuclear cells. B, C. Luciferase activity of promotor assay vectors. In the mutant the corresponding sites of the two strands of the DNA were mutated from A to G. Promotor activities (corrected for transfection efficiency) are presented as fold increase relative to empty vector (PGL4.20). The fold ratio of empty PGL4.20 Firefly Luciferase plasmid to PGL4.73 Renilla transfection control vector was normalized to 1. Mean ± SD, n = 3 independent experiments.

The *ATG5* rs510432 SNP is located upstream of *ATG5’s* first exon in the putative promotor region. Thus, we examined the potential functionality of this SNP. Human genomic DNA was isolated from peripheral blood mononuclear cells and was subjected to PCR amplification in order to generate four fragments of different lengths containing the Atg5 promotor (Figure 3). All promotor fragments resulted in enhanced luciferase activity. Maximal activity was shown for fragments 2,417 bp and 3,239 bp (–3555 to –377 and –2749 to –377, respectively). Larger fragments containing upstream sequences (–6081 to –377 and –4532 to –377, respectively) resulted in relative reduction of promotor activity, suggesting the presence of upstream regulatory elements. Because fragment 3,239 bp represented the largest promotor fragment with maximal activity, this fragment was used as a template for introduction of base change from A to G (asthma genotype) at rs510432. The variant “G” allele resulted in significantly enhanced promotor activity by 23% compared to the non-variant “A” allele (p<0.007*)* (Figure 3B). Further, we queried the TRANSFAC database for transcription factor binding sites which include rs510432 and identified two transcription factors, STAT1 and C-Fos, which have been associated with asthma.

### Atg5 mRNA Expression Increased in Acute Asthma

**Figure 4:**
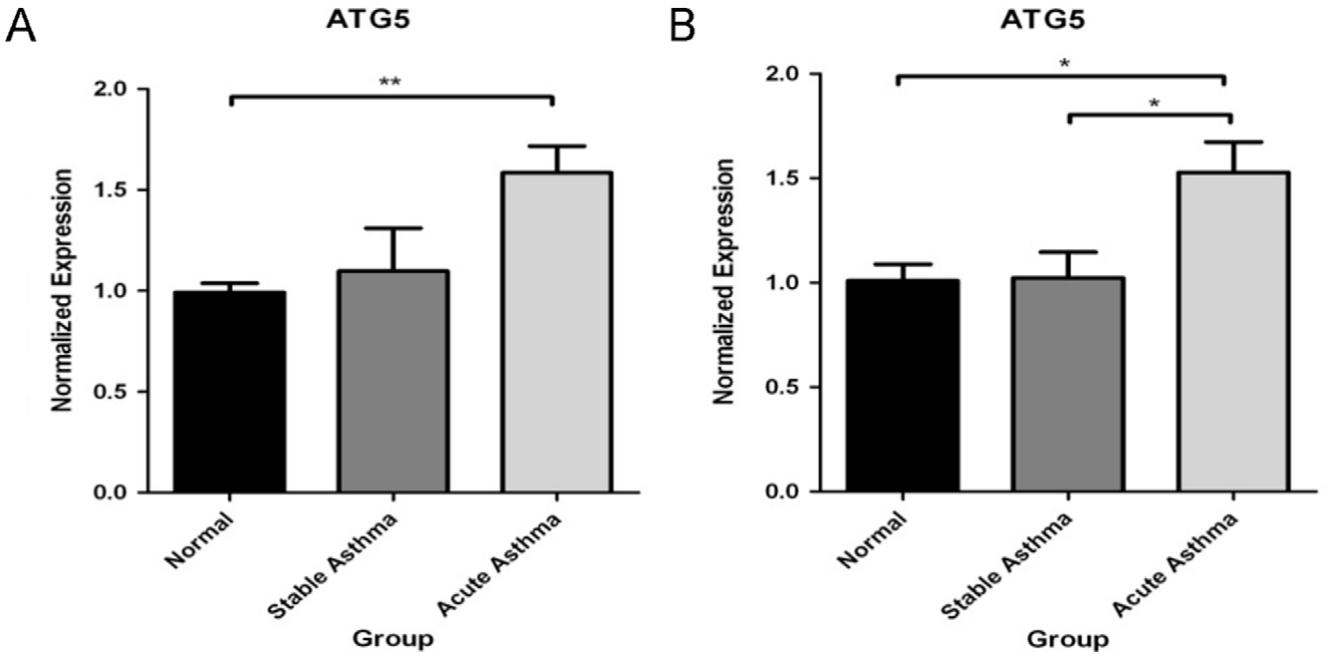
ATG5 expression enhanced in nasal mucosal samples from children with acute asthma. Data are presented as normalized expression from Affymetrix array data using (A) 202511_s_at and (B) 210639_s_at *ATG5* probesets. Intensities were normalized to the median expression level of the control samples.

Based on our results thus far, we hypothesized that *ATG5* expression would be increased in patients with asthma. Therefore, we compared *Atg5* gene expression in nasal mucosal cells (>92% airway epithelial cells) isolated from children with acute or stable asthma, as well as from non-asthmatic control children. *ATG5* expression was significantly increased in the nasal cells derived from children with acute asthma compared to non-allergic controls with two distinct probe sets (202511_s_at, p = 0.0057; 210639_s_at, p = 0.021) (Figure 4). Notably, stable asthmatics had an intermediate expression level, but were not significantly different from controls.

## Discussion

Our data support a role for the autophagy pathway, and specifically *ATG5* but not *ATG7*, in childhood asthma. We identified two variants in the *ATG5* gene that are associated with asthma using a genetic association study and uncovered additional novel associations using genotypes inferred through imputation. We investigated the biologic relevance of the *ATG5* rs510432 SNP and found that the disease-associated allelic variant confers enhanced *ATG5* promotor activity. When we queried the TRANSFAC database for transcription factor binding sites that include rs510432, we identified two transcription factors, STAT1 and C-Fos, which have been associated with asthma and may be mediating the functional differences observed. Further, we demonstrated that *Atg5* mRNA expression is up-regulated in human nasal epithelial cells during an acute asthma attack. Collectively, our results suggest a novel function for *ATG5* in asthma.

By screening the autophagy genes *ATG5* and *ATG7*, we found evidence of association with variants in *ATG5* with childhood asthma. This is a novel finding as there have not been any reported associations of *ATG5* as a candidate gene for asthma based on searches using PUBMED (terms *ATG5* genetic association, date 8/29/2011) and the National Human Genome Research Institute’s Catalog of Published Genome-Wide Association Studies (http://www.genome.gov/gwastudies/index.cfm?pageid=26525384#searchForm assessed 8/29/2011) [26]. One of our primary findings was that the promotor variant rs510432 was associated with childhood asthma in the GCPCR cohort, where asthmatics had a 1.47 fold increase of having the minor allele G compared to controls. To determine whether this promotor variant had functional effects, the promotor activity of *ATG5* variants with the A and G alleles were compared. The G allele had higher promotor activity than the A allele. The increased promotor activity of the allele associated with increased risk of asthma (G) is consistent with our gene expression studies showing increased gene expression of *ATG5* in asthmatics. Further, the increased promotor activity is consistent with the previous study demonstrating increased activation of autophagy proteins in lung tissue from chronic obstructive pulmonary disease patients [10].

Several other *ATG5* SNPs were found to be associated with childhood asthma. The genotyped *ATG5* rs12201458 SNP is located in intron 7, the last intron in the gene. This SNP is predicted to be an intronic enhancer with a low predicted risk of functional effects using FASTSNP [27]. As, the SNPs genotyped for *ATG5* were predominantly tagging, the identified association with rs122201458 may be due to linkage disequilibrium with functional variants. Thus, the genotypes at markers that had not been genotyped in this study were imputed using a reference panel. Imputation can permit the comparison of studies which focused on different SNPs. Using meta-analysis, four SNPs exhibited significant association across the studies.

We found *Atg5* expression to be up-regulated in nasal epithelium from acute asthmatics. While there have been no previous studies investigating *Atg5* expression in asthmatics, there is evidence of increased expression of autophagic proteins, including *Atg5*, in lung tissue from patients with chronic obstructive pulmonary disease [10]. The respiratory tract is divided into the upper airway (UA; the portion from the nose to the vocal cords) and the lower airway (LA; below the vocal cords). Epidemiologic and biologic evidence support this concept of a “united airway” in which the UA reflects pathophysiologic changes occurring in the LA and vice versa through biological cross-talk. Studies have demonstrated that comparable inflammatory processes underlie rhinitis and asthma [28], [29]; and not only does nasal allergen challenge initiate pulmonary inflammation [30], but lung allergen challenge induces inflammation in bronchial and nasal mucosa [31]. The autophagy pathway is responsive to cigarette smoke exposure [32] and viral infection [9], [33], important cofactors for asthma, further supporting a role of autophagy in respiratory disease.

While, *Atg5* is necessary for antigen presentation [8] and can lead to increased viral clearance [34], autophagy machinery can also be hijacked to increase viral replication [33]. *Atg5*, though indispensible for autophagy, has functions independent of autophagy including a role in apoptosis and regulation of interferon (IFN) responses against viral infections [9], [35]. Indeed, the *Atg12-Atg5* conjugate has been shown to negatively regulate the type I IFN modulating pathway [9]. Thus, in contrast to anti-pathogenic properties of autophagic processes, *Atg5* also has the capacity to promote RNA virus replication by inhibiting innate anti-virus immune responses, a rather paradoxical role for *Atg5*. These non-canonical roles for *Atg5* in regulation of apoptosis and IFN production could have significance in asthma pathology in relation to immune responses to viral infections. Our data has demonstrated amplified *Atg5* expression in acute asthmatics. Consistent with previous studies indicating that asthmatics have slower viral clearance [36], increased *Atg5* expression could lead to augmented viral replication, greater virus production and thus prolonged viral clearance. Taken together, these studies provide a potential mechanism for a role of ATG5 in asthma. Future studies are required to determine if ATG5 has a causal role in asthma or if these differences are due to inflammation and related cell death.

A major strength of our study is the use of functional investigations to complement the genetic associations. Indeed, many genetic association studies have reported associations with asthma, but few have characterized functional effects [37]. Our data provide strong evidence for association of a functional promotor SNP in *ATG5* with childhood asthma. Our study is limited in that we utilized adult controls in one of the cohorts. Although many studies prefer to match cases and controls on age, studies have utilized adults as controls for childhood asthma as they represent a truly asthma-free population, whereas similarly aged child controls may go on to develop asthma [38]. While this has a potential to create bias, this cohort was used only to confirm findings and therefore the risk of incorrect inference is minimal. Some of the cohorts were of modest size, but it is important to note that both the CAMP and CARE cohorts have extremely well characterized subjects with detailed phenotypic data. However, replication in additional cohorts is warranted.

In summary, several independent investigators have linked autophagy to various aspects of the innate and adaptive immunity. *ATG5* is a key component of the autophagy machinery and has functions in viral clearance. We have demonstrated that *ATG5* variants are associated with childhood asthma, including a variant that confers enhanced promotor activity and that *Atg5* expression is dysregulated in children with asthma. Additional studies are necessary to further elucidate biological roles of autophagy and autophagy-related antiviral defense in asthma pathogenesis.
